# Supplementary material for: Identification of Cancer Stem Cell Subpopulations in Head and Neck Metastatic Malignant Melanoma
Source: Cells. 2020 Jan 30;9(2):324. doi: 10.3390/cells9020324 (PMC7072148; doi:10.3390/cells9020324)
Supplement: Supplementary file 1 [file cells-09-00324-s001.pdf]

cells

MDPI

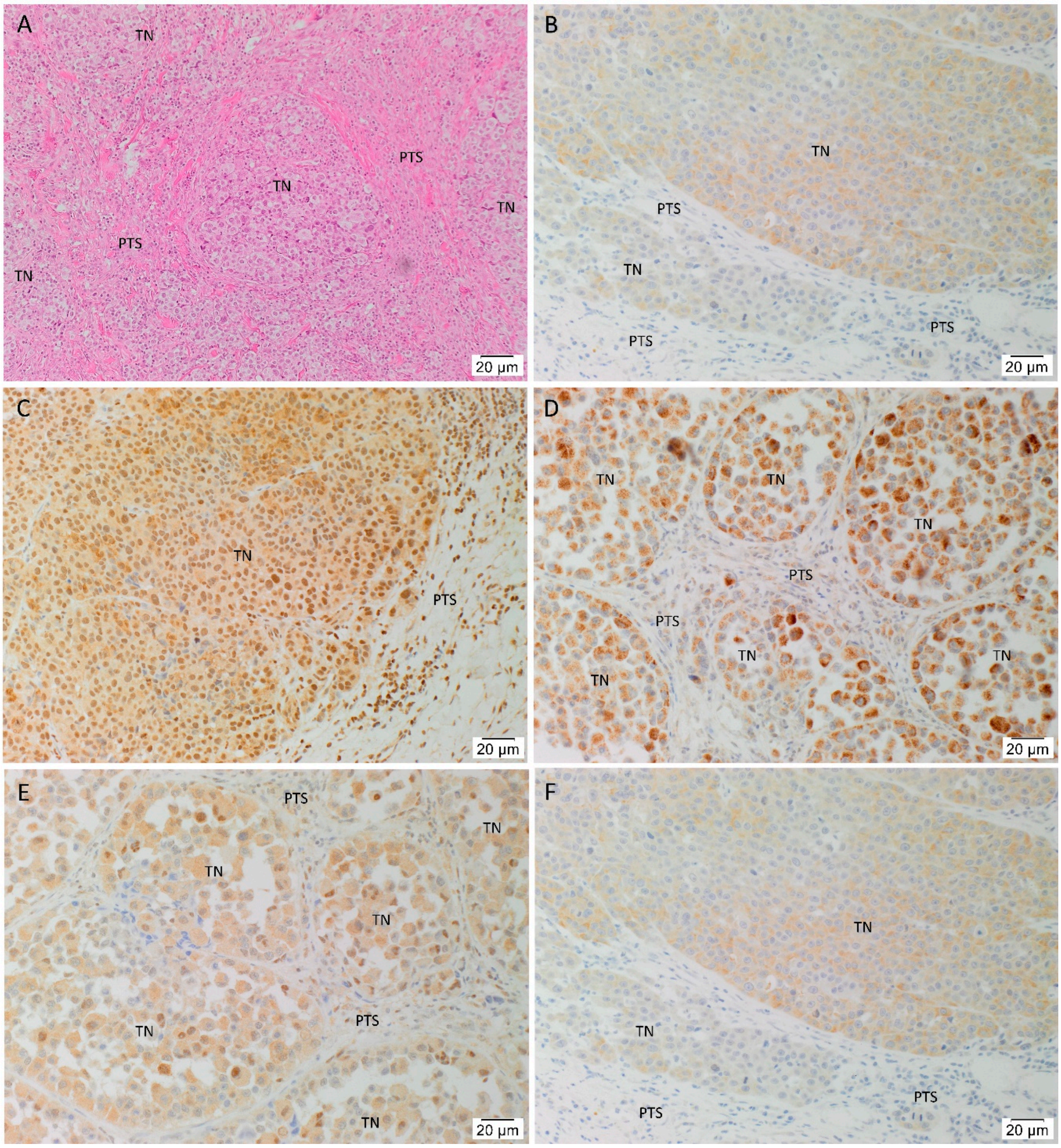

Figure S1. Representative low magnification images of hematoxylin and eosin (**A**) and immunohistochemical (**B**–**F**) staining of head and neck metastatic malignant melanoma (HNmMM) demonstrating the tumor nests (TNs) surrounded by peritumoral stroma (PTS), and the expression of OCT4 (**B**, brown), SOX2 (**C**, brown), KLF4 (**D**, brown), c-MYC (**E**, brown) and NANOG (**F**, brown). Nuclei were counterstained with hematoxylin (**A**–**F**, blue). Original magnification 200×; $n = 20$.

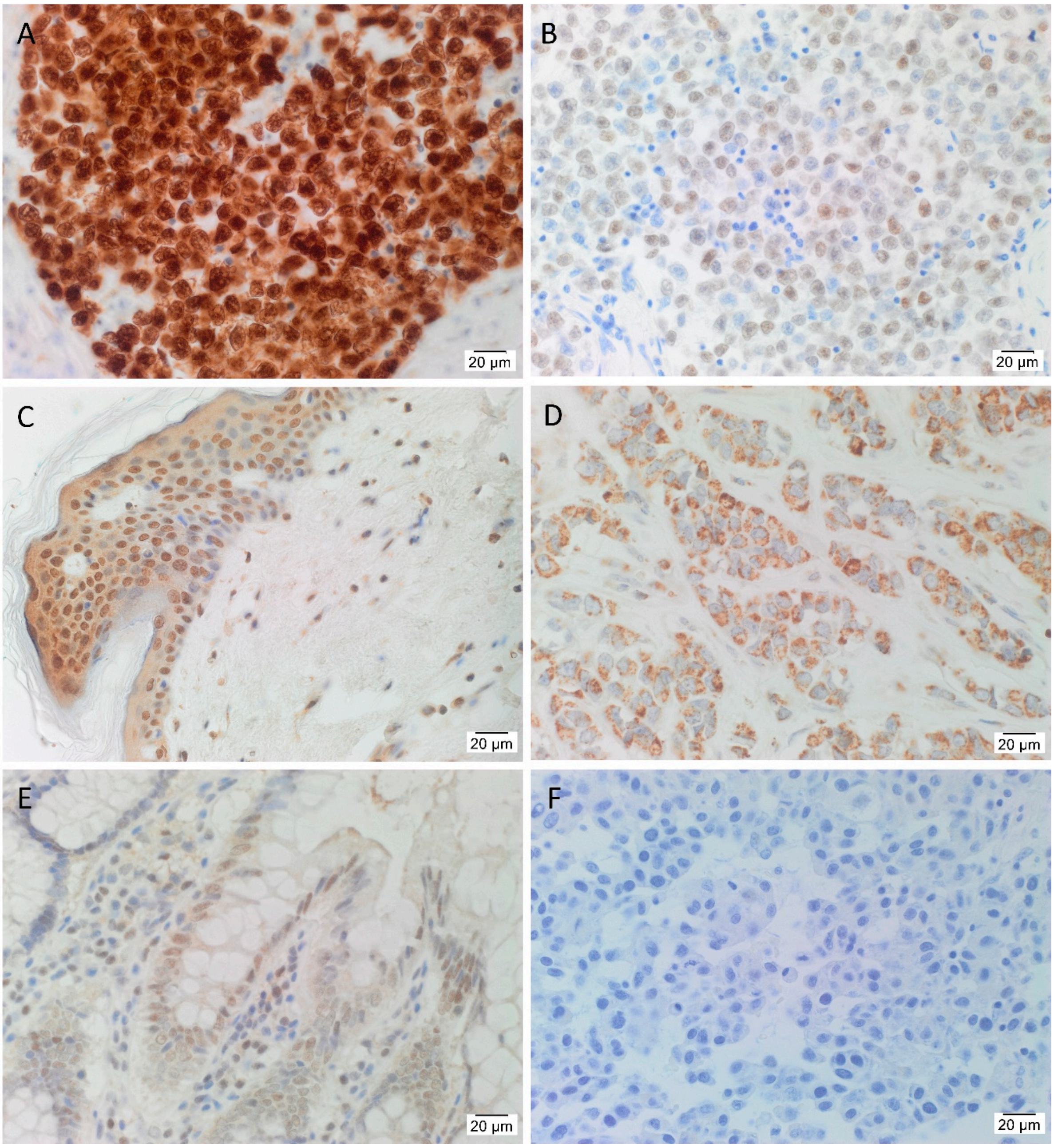

Figure S2. Representative immunohistochemical stained sections of positive and negative human control tissues for induced pluripotent stem cell markers showing expected staining pattern for OCT4 (**A**, brown) and NANOG (**B**, brown) in seminoma, SOX2 (**C**, brown) in normal skin, KLF4 (**D**, brown) in breast carcinoma, and c-MYC (**E**, brown) in normal colon tissue. Negative controls were performed on a section of head and neck metastatic malignant melanoma tissue sample using a matched IgG isotype control for rabbit primary antibody (**F**). Nuclei were counterstained with hematoxylin (**A**–**F**, blue). Original magnification: 400×.

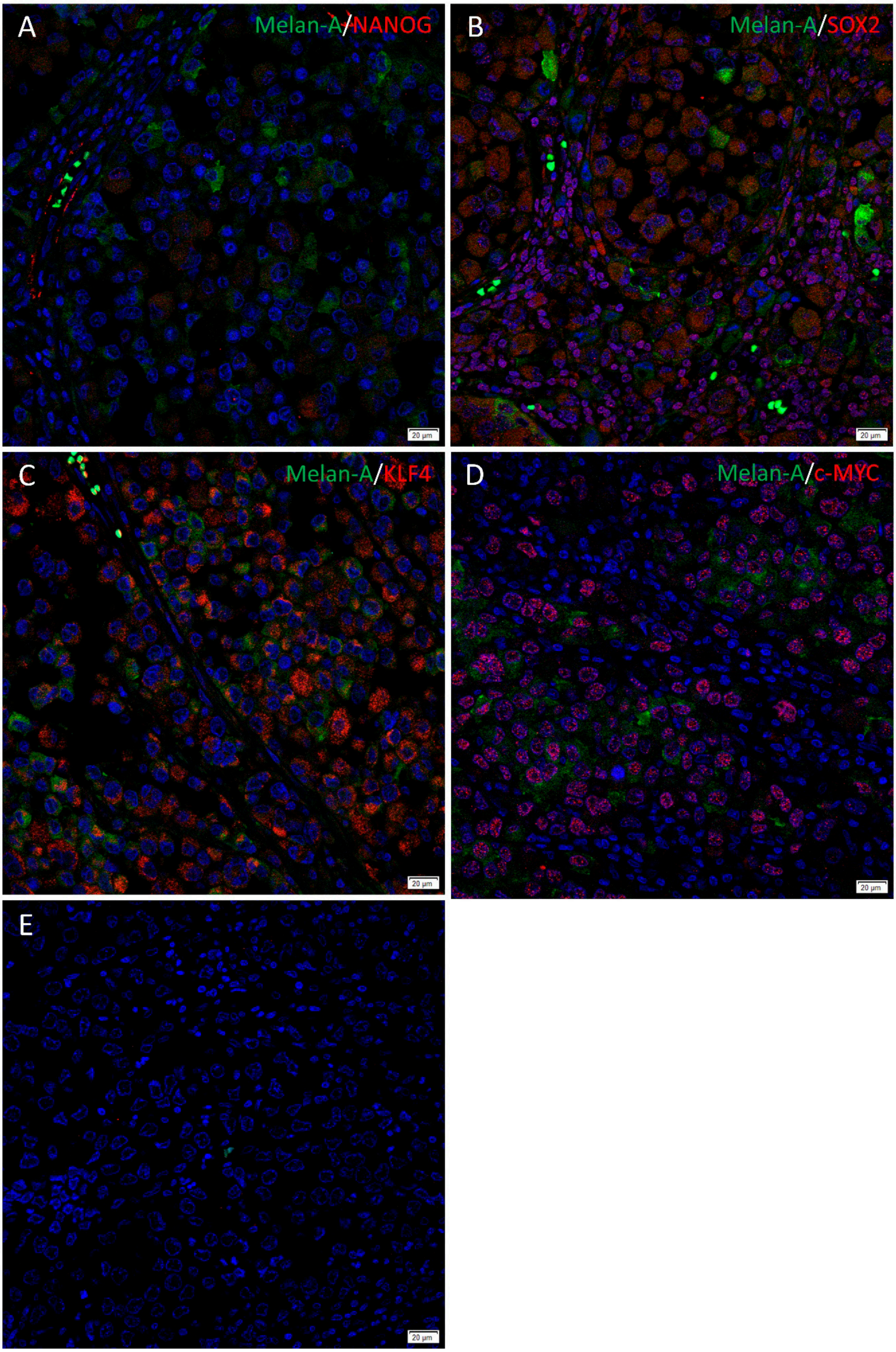
A
Melan-A/NANOG
B
Melan-A/SOX2
C
Melan-A/KLF4
D
Melan-A/c-MYC
E
20 μm

Figure S3. Representative images of immunofluorescence dual-staining of head and neck metastatic malignant melanoma (HNmMM) tissue samples for the melanoma marker Melan-A and induced pluripotent stem cell markers showing expression of NANOG (**A**, red), SOX 2 (**B**, red), KLF4 (**C**, red) and c-MYC (**D**, red) on some Melan-A+ (**A**–**D**, green) cells within the tumor nests and some cells within the peritumoral stroma. A negative control of a HNmMM demonstrated minimal staining (**E**). Cell nuclei were counterstained with 4′,6′-diamidino-2-phenylindole (**A**–**E**, blue). Original magnification: 400×.

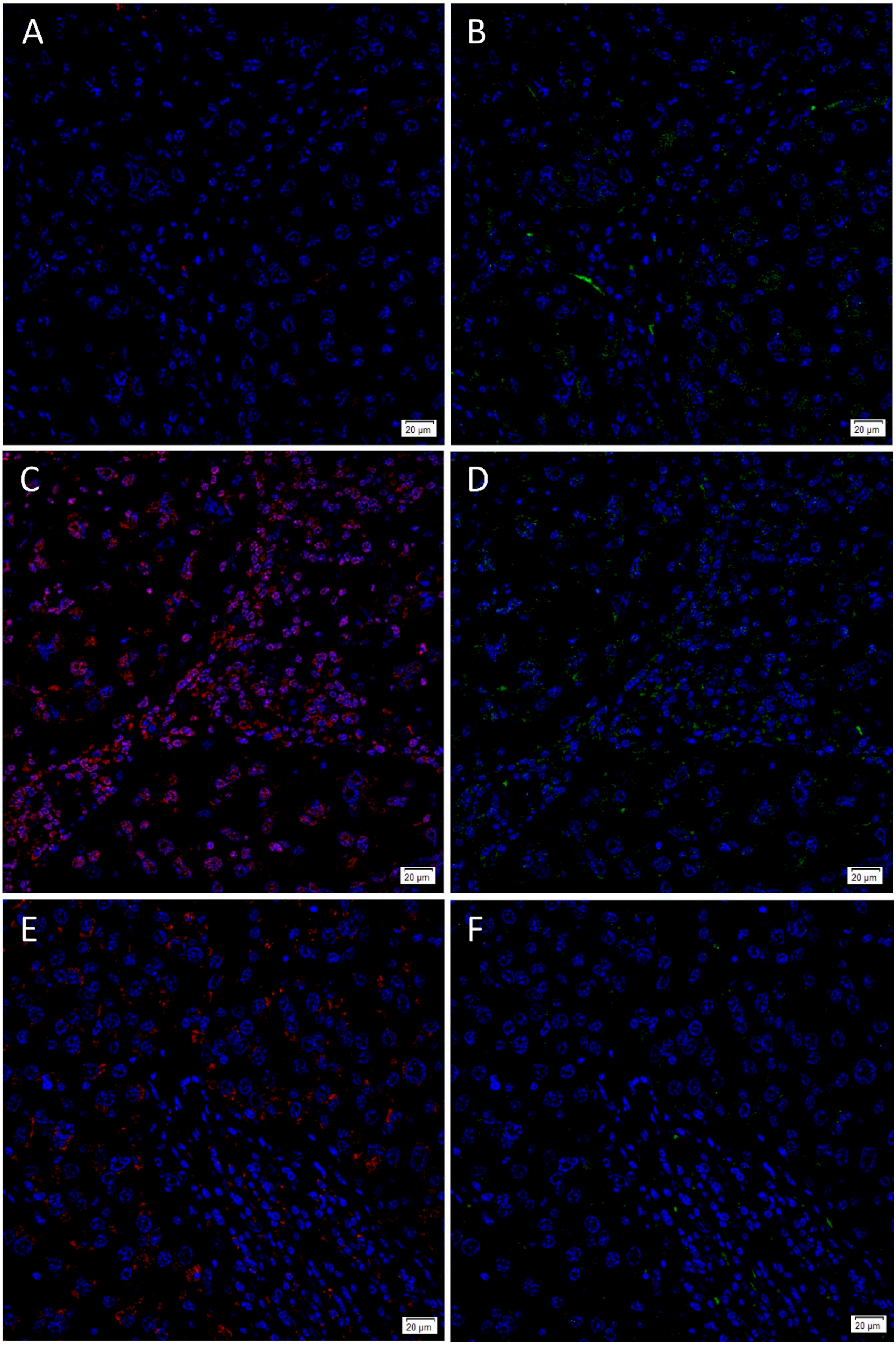
A
B
C
D
E
F
20 μm
20 μm
20 μm
20 μm
20 μm
20 μm

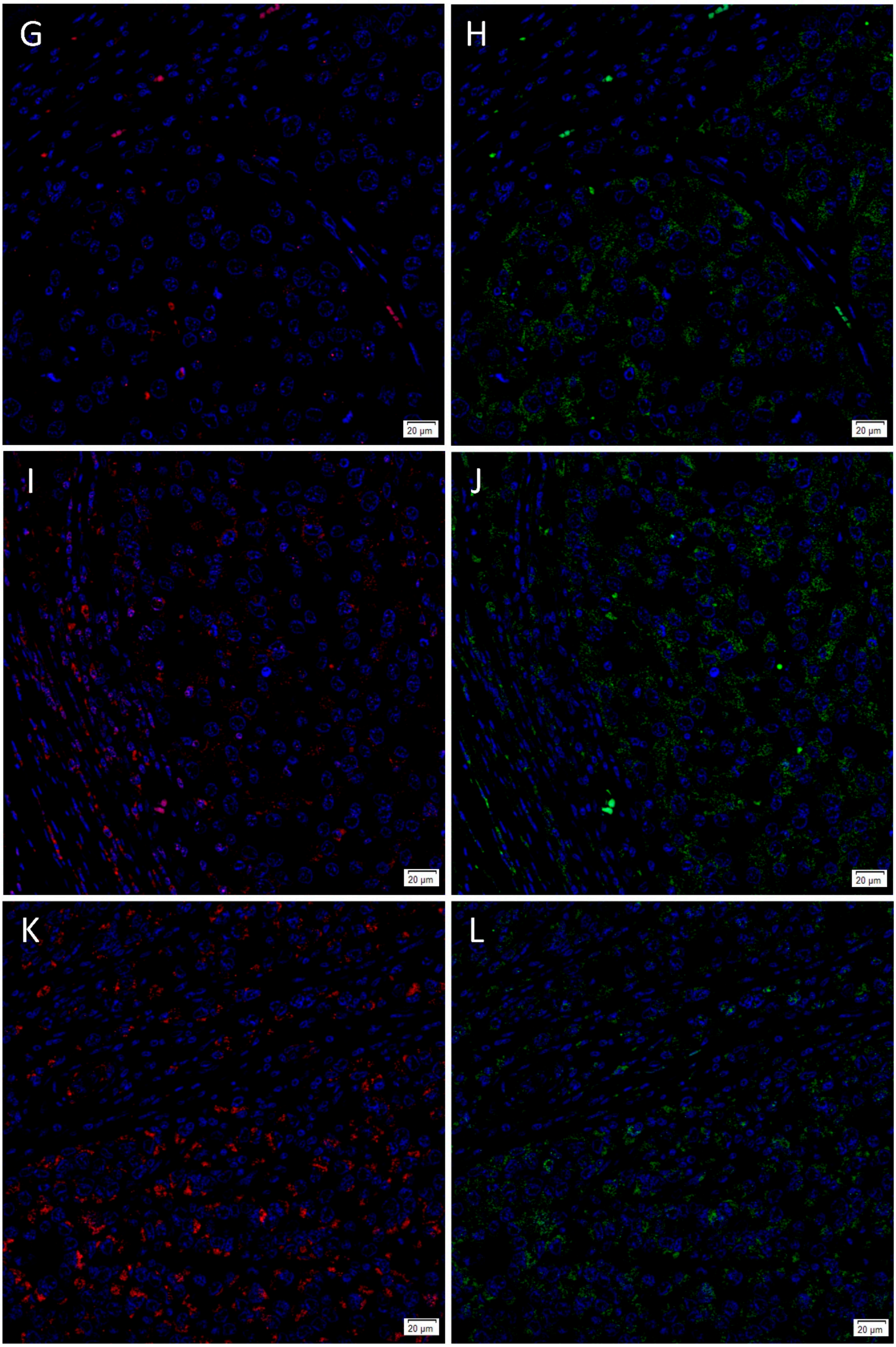
G
H
I
J
K
L
20 µm

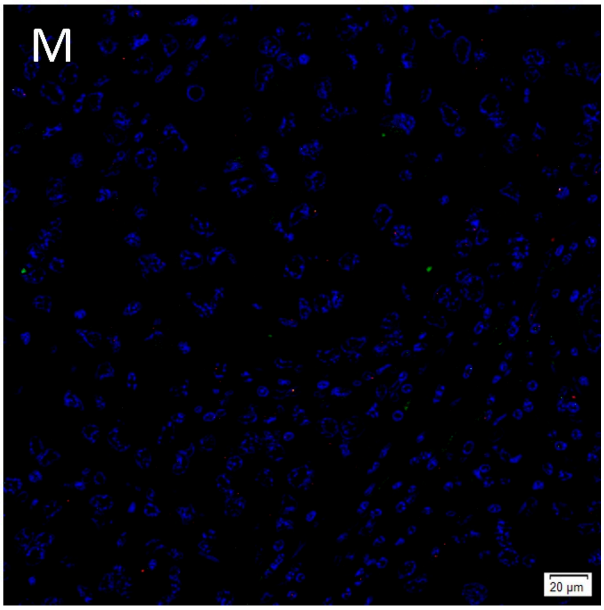

Figure S4. Split immunofluorescence immunohistochemical stained images of head and neck metastatic malignant melanoma tissue samples shown in Figure 3. Expression of NANOG (**A**, red) and OCT4 (**B**, green); SOX2 (**C**, red) and OCT4 (**D**, green); KLF4 (**E**, red) and OCT4 (**F**, green); NANOG (**G**, red) and c-MYC (**H**, green); SOX2 (**I**, red) and c-MYC (**J**, green); KLF4 (**K**, red) and c-MYC (**L**, green); and a negative control (**M**) are demonstrated. Cell nuclei were counterstained with 4′,6′-diamidino-2-phenylindole (**A**-**M**, blue). Original magnification: 400×.

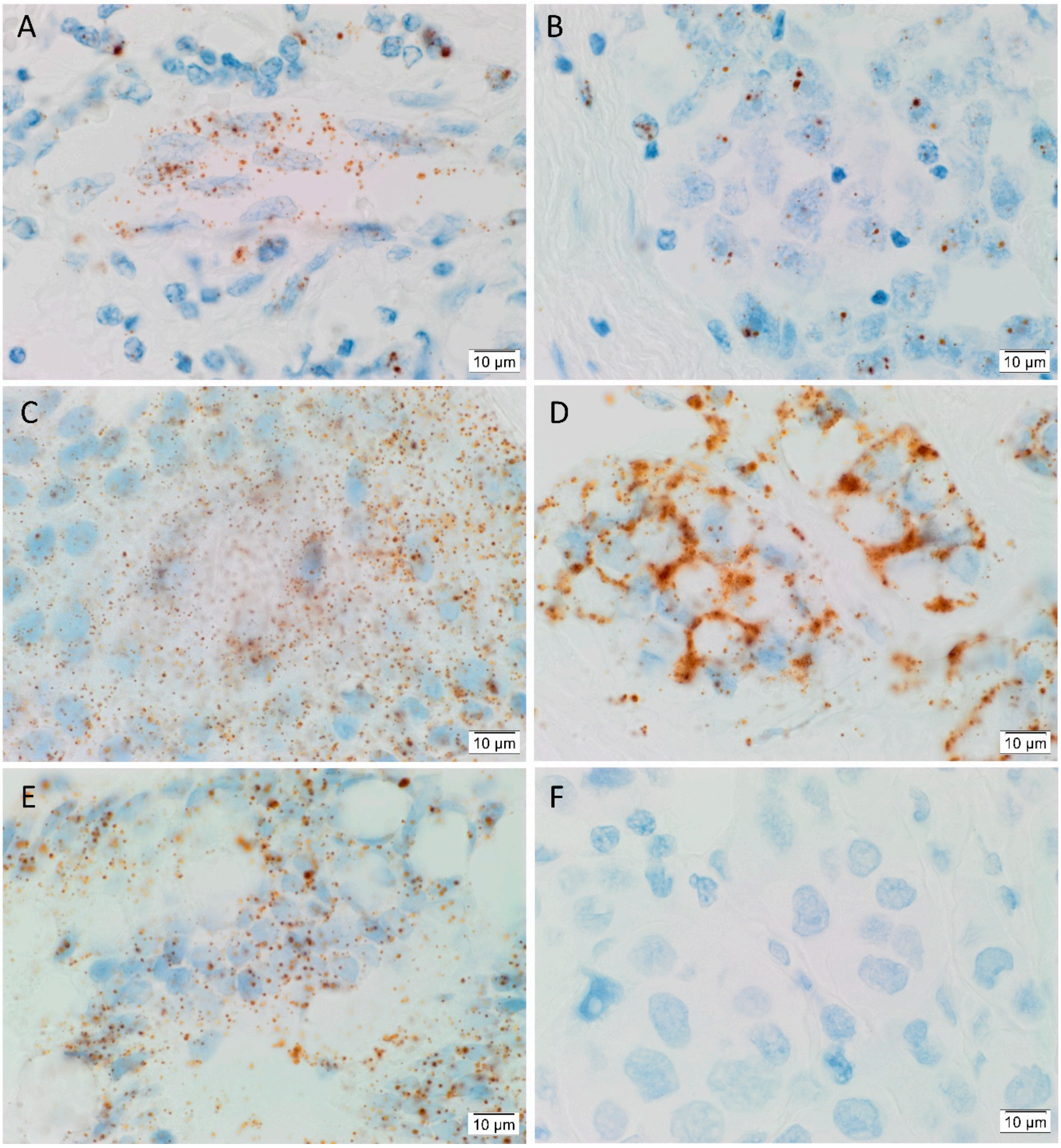

Figure S5. Positive and negative controls of in situ hybridization stained sections for the five induced pluripotent stem cell markers. Positive control section of human seminoma for OCT4 (**A**, brown) and NANOG (**B**, brown), normal skin for SOX2 (**C**, brown), breast carcinoma for KLF4 (**D**, brown) and normal colon tissue for c-MYC (**E**, brown). Negative control (**F**) showed no background activity. Nuclei were counterstained with hematoxylin (**A**–**F**, blue). Original magnification 1000×.

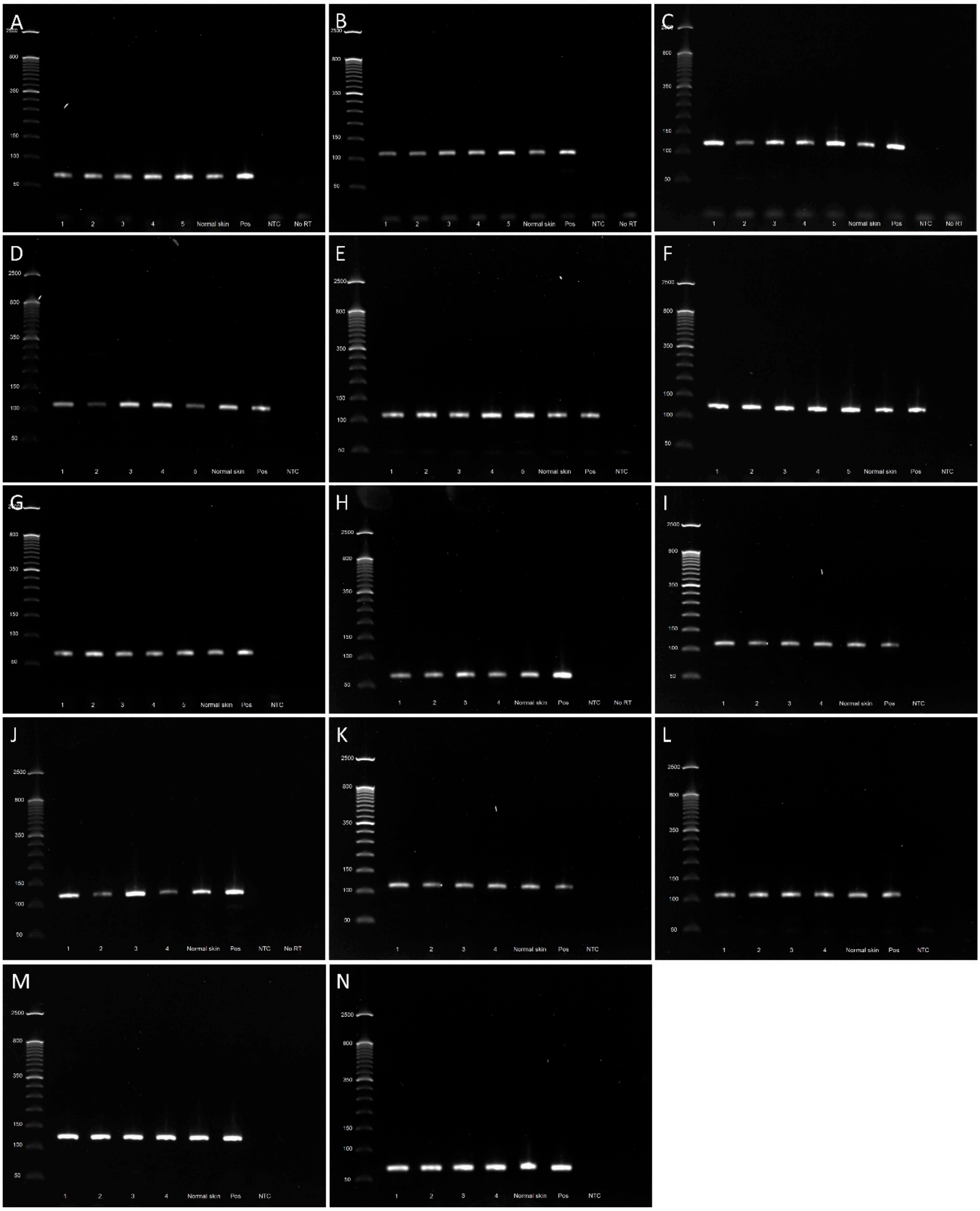

Figure S6. Reverse transcriptase quantitative polymerase chain reaction (RT-qPCR) amplification products from five head and neck metastatic malignant melanoma (HNmMM) tissue samples (**A**–**G**) and four HNmMM-derived primary cell lines (**H**–**N**) were checked using agarose gel electrophoresis. Five HNmMM tissue replicate qPCR amplification products were probed for OCT4 (**A**, 64bp), NANOG (**B**, 109bp), SOX2 (**C**, 91bp), KLF4 (**D**, 110bp) and c-MYC (**E**, 107bp). Four HNmMM-derived primary cell lines qPCR amplification products were probed for OCT4 (**H**, 64bp), NANOG (**I**, 109bp), SOX2 (**J**, 91bp), KLF4 (**K**, 110bp) and c-MYC (**L**, 107bp). The housekeepers, GAPDH (122bp) and PSMB4 (63bp), from HNmMM tissue samples (**F**) and (**G**) and cell lines (**M**) and (**N**) were also checked. Ladder refers to the DNA marker in base pairs (bp); Lanes 1–5 refer to the respective tissue/cell samples; Pos, positive control (NTERA2 cell lines); NTC, no template control (RNase-free

water) to confirm no contamination; No RT, Reverse transcriptase negative control for primers that may detect genomic DNA.

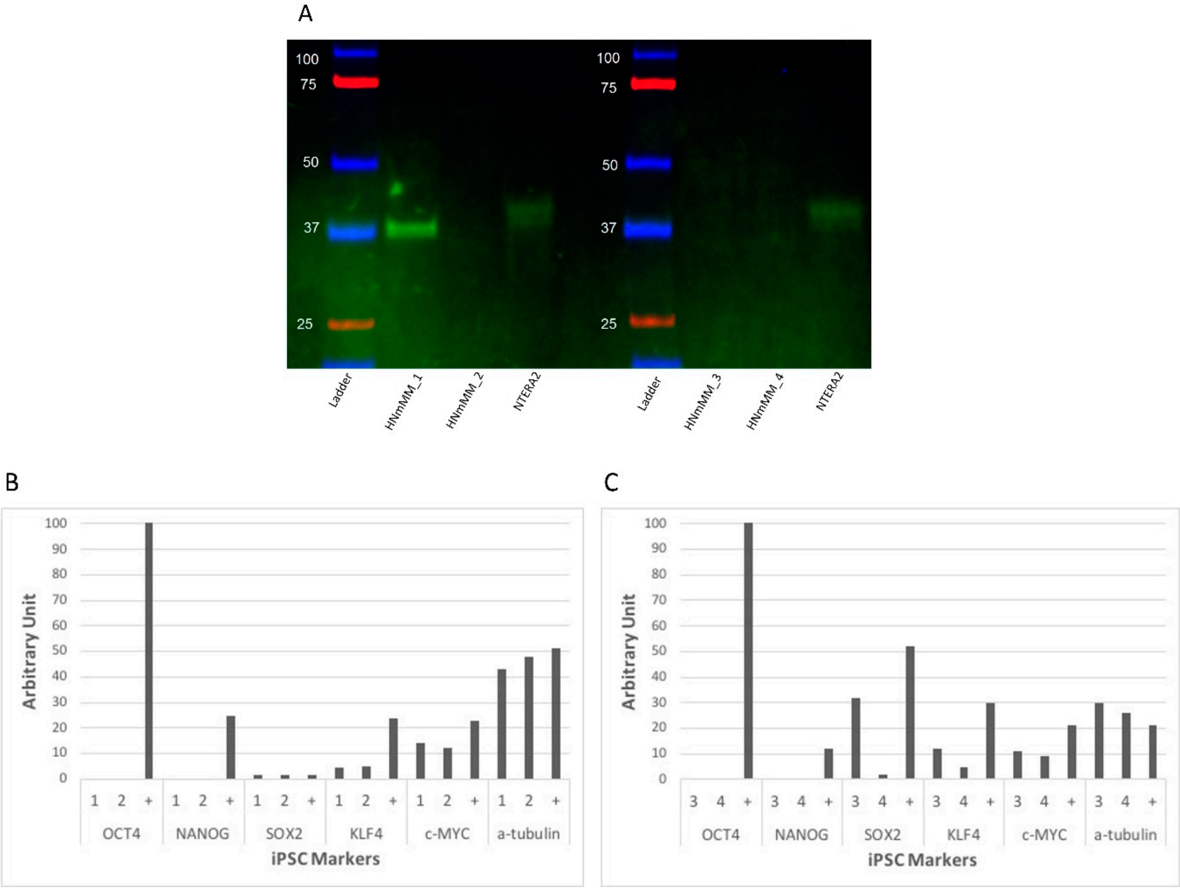

Figure S7. Western blot IgG isotype matched control (**A**) and densitometric quantification of induced pluripotent stem cell biomarker signals from four head and neck metastatic malignant melanoma (HNmMM) cells (1–4) run on western blots (**B**,**C**). In Panel B, 1, 2 refer to HNmMM cell lines 1 and 2 and in Panel C, 3, 4 refer to HNmMM cell lines 3 and 4, respectively. + refers to the positive control; the values are relative to α-tubulin levels from each blot. OCT4 and NANOG show negligible level of expression across the two cell lines, while SOX2 expression levels are low overall apart from one cell line. KLF4 and c-MYC expression across the cell lines are comparable. In Panel A, 19-024, 19-057, 18-245, and 19-172 refer to the four cell lines and Ntera2, the positive control.

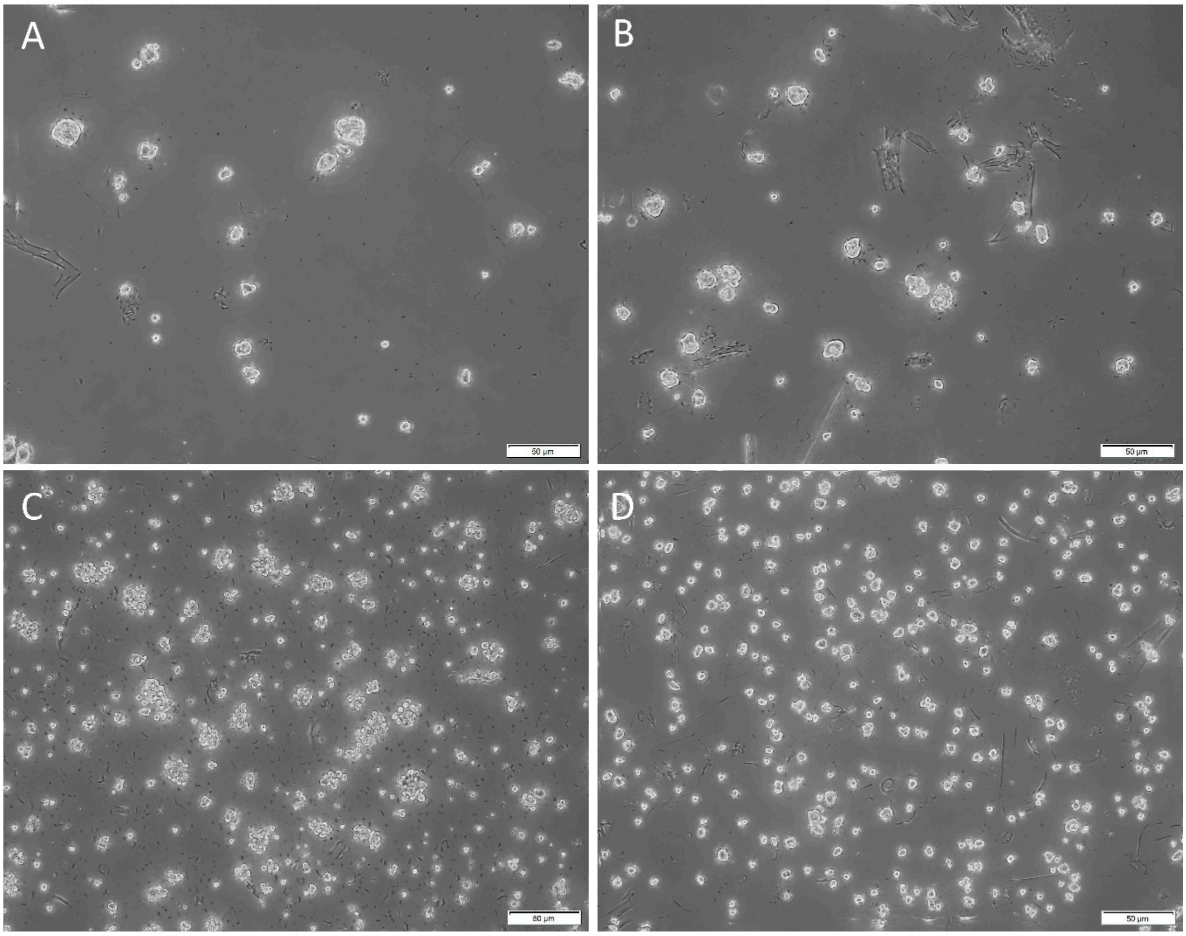

Figure S8. Representative low magnification (10×) z-stack images of tumorsphere formation in suspension culture of the three head and neck metastatic malignant melanoma-derived primary cell lines (**A**–**C**). A representative low magnification (10×) image of one HNmMM-derived primary cell line that did not form spheres, as defined by the tumorsphere criteria in Methods (**D**).

Table S1. Demographic data and characteristics of the metastasis of the 20 patients with head and neck metastatic malignant melanoma.

Patient	Gender	Age (years)	Metastatic Site
1	M	103	CLN
2	M	77	CLN
3	F	83	CLN
4	F	67	CLN
5	M	56	CLN
6	M	73	CLN
7	M	70	PLN
8	M	73	CLN
9	M	70	CLN
10	F	86	CLN
11	M	78	PLN
12	M	66	CLN
13	F	79	PLN
14	M	76	CLN
15	M	78	PLN
16	M	77	CLN
17	M	47	CLN
18	M	64	CLN
19	M	59	CLN
20	M	83	PLN

M, male; F, female; PLN, parotid lymph node. CLN, cervical lymph node.

© 2019 by the authors. Submitted for possible open access publication under the terms and conditions of the Creative Commons Attribution (CC BY) license (http://creativecommons.org/licenses/by/4.0/).
